# Potential sources of pain in symptomatic degenerative meniscal tear: A narrative review

**DOI:** 10.1016/j.ocarto.2025.100616

**Published:** 2025-04-24

**Authors:** Love Tsai, Elizabeth Matzkin, Morgan H. Jones, Rachel E. Miller, Jeffrey N. Katz

**Affiliations:** a Orthopedic and Arthritis Center for Outcomes Research, Department of Orthopedic Surgery, Brigham and Women's Hospital, Boston, MA, USA; b Department of Orthopedic Surgery, Brigham and Women's Hospital, Boston, MA, USA; c Division of Rheumatology, Inflammation and Immunity, Brigham and Women's Hospital, Boston, MA, USA; d Department of Epidemiology, Harvard Chan School of Public Health, Boston, MA, USA; e Harvard Medical School, Boston, MA, USA; f Division of Rheumatology, Rush University Medical Center, Chicago, IL, USA

**Keywords:** Degenerative meniscal tear, Knee osteoarthritis, Knee pain, Mechanical symptoms

## Abstract

**Importance:**

Although degenerative meniscal tear is relatively prevalent in older persons, the sources of the accompanying pain and mechanical symptoms—such as knee clicking, locking, and popping—are unclear, making targeted treatment difficult. We conducted a narrative literature review to synthesize research surrounding sources of symptomatic degenerative meniscal tear.

**Observations:**

We identified five mechanisms of symptom generation in patients with degenerative meniscal tear: obstruction, abnormal load bearing, inflammation, neoinnervation, and central sensitization. We confirmed clinical observations that degenerative meniscal tear often occurs concomitantly with knee osteoarthritis, which adds complexity to symptom attribution.

**Conclusions and relevance:**

Degenerative meniscal tear may lead to pain or mechanical symptoms through a variety of pathways. In identifying the diversity in pathways and corresponding physiological changes leading to symptomatic degenerative meniscal tear, this review may help clinicians contextualize the condition and target therapies for their patients.

## 1 Introduction

Degenerative meniscal tear is commonly observed in imaging studies of middle-aged and older adults. In 2008, the prevalence of a right knee meniscal tear observed on MRI among community-dwelling persons between the ages of 50 and 90 years was 31 ​% (95 ​% confidence interval (CI) [28 ​%, 34 ​%]) [1]. Conventional teaching has emphasized that meniscal tears may present as pain localized to the joint line as well as mechanical abnormalities such as clicking, catching, popping, locking, and giving way [2]. However, most participants (61 ​%) with meniscal tear in this study did not exhibit *any* symptoms at imaging. It is unclear what processes lead to symptoms in persons with degenerative meniscal tear. Mechanical, load-based, inflammatory, neoinnervation, and sensitization mechanisms have all been proposed in various contexts and with varying degrees of confidence. “Mechanical” refers to symptoms stemming from disruption of normal smooth knee motion by the torn meniscal tissue while “load-based” refers to discomfort generated by the increased forces on surrounding knee structures. “Inflammatory,” “neoinnervation,” and “sensitization” processes reflect broader physiological changes that may accompany such an injury—namely the increase of tissue inflammation, pain-sensitive nerves, and abnormal pain processing. In this paper, we revisit the proposed biomechanical sources of symptoms in older persons with degenerative meniscal tear and discuss new findings that contextualize our understanding of these five mechanisms.

### 1.1 Meniscal tear

**Fig. 1:**
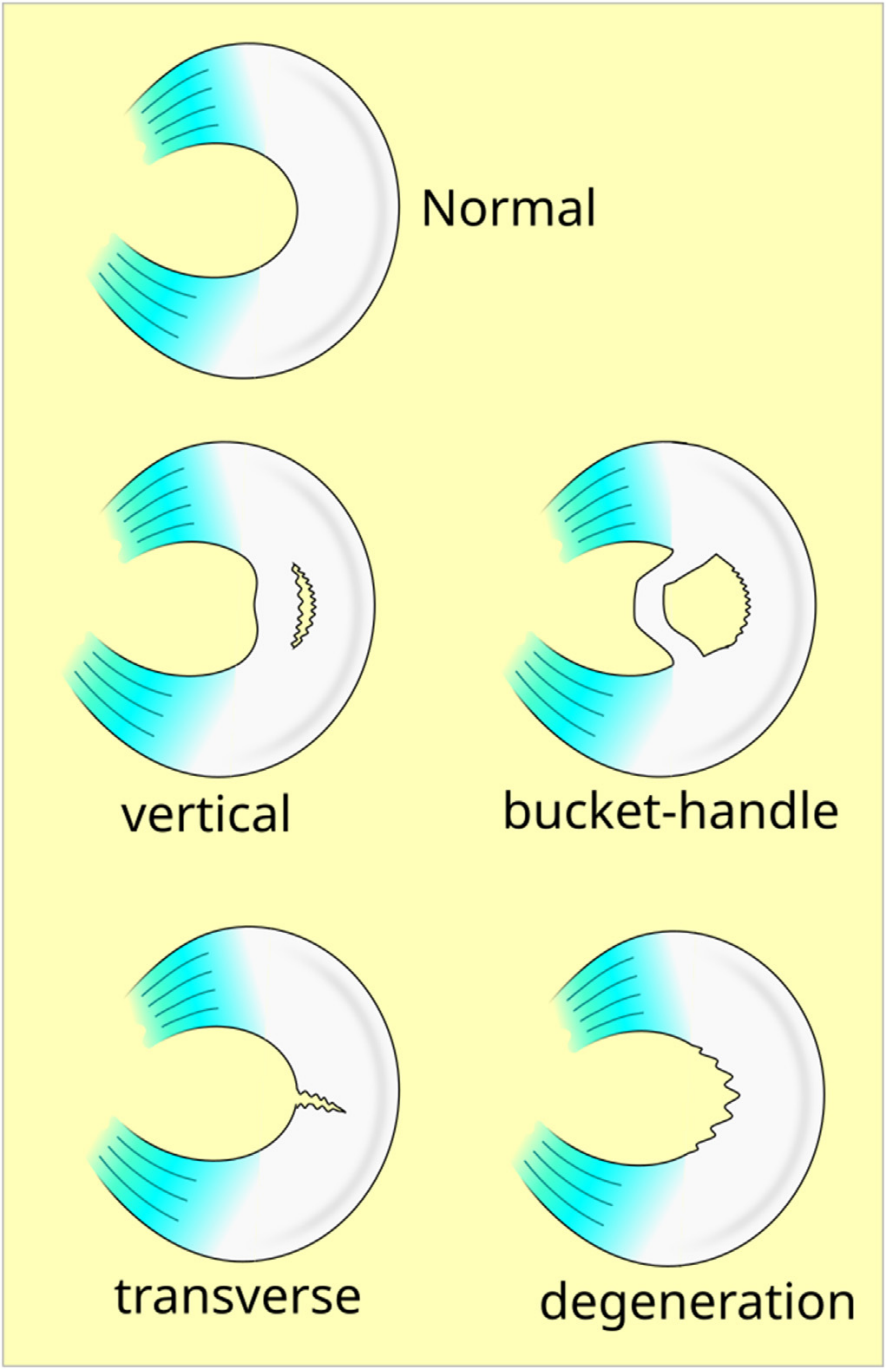
Meniscal Tear Morphologies (Illustrated). Karia, M., Ghaly, Y., Al-Hadithy, N. et al. Current concepts in the techniques, indications and outcomes of meniscal repairs. Eur J Orthop Surg Traumatol 29, 509–520 (2019). https://doi.org/10.1007/s00590-018-2317-5.

**Fig. 2:**
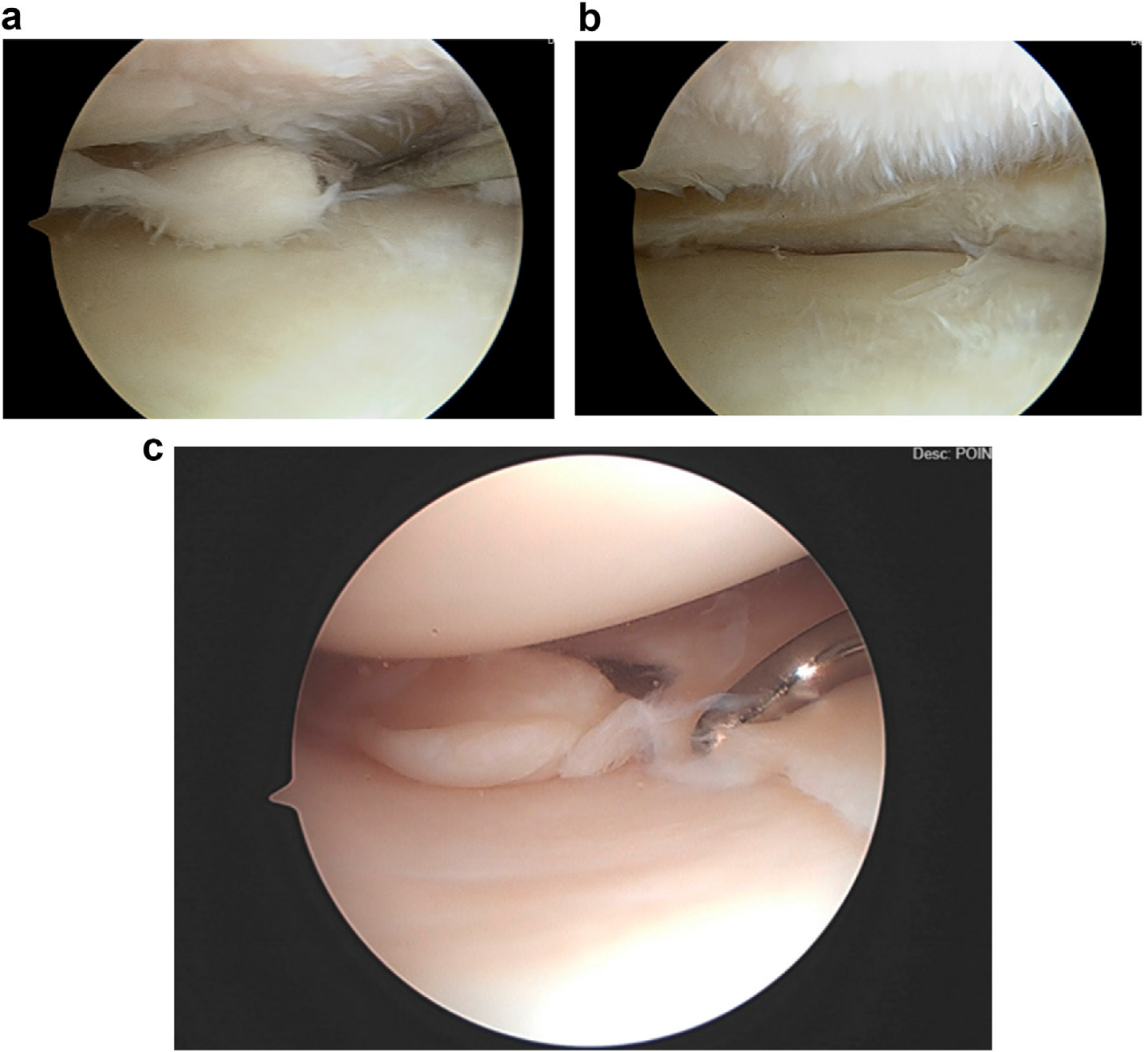
a. Arthroscopic image of the medial compartment of a right knee demonstrating a complex degenerative medial meniscal tear with knee OA (Outerbridge Grade II/III changes of the medial femoral condyle). b. Arthroscopic image of the same right knee medial compartment after partial medial meniscectomy. c. Arthroscopic image of the medial compartment of a right knee demonstrating an oblique medial meniscal tear. This type of tear has the potential to cause meniscal mechanical symptoms such as catching or locking (obstruction).

The menisci are a pair of semilunar, wedge-shaped cartilaginous structures positioned between the femoral condyles and tibial plateau [3]. Meniscal tears can be either traumatic or degenerative and occur anywhere in the menisci, in a range of tear patterns (Fig. 1, Fig. 2) [4]. Traumatic tears occur more often in younger persons, typically in association with an event that creates excessive shear force (such as a sporting injury, fall, or traffic accident) [5,6]. Degenerative tears, on the other hand, do not arise from acute trauma, but rather from normal forces exerted repeatedly upon an aging knee or chronic overloading upon a normal knee [7]. These tears are typically seen in adults older than 40 years, as well as in patients with knee osteoarthritis (OA) [8]. Rather than exhibiting any one tear pattern, degenerative tears often exhibit multiple tear patterns and are known as “complex” tears [9]. The relative simplicity of traumatic tear morphology makes meniscal repair the recommended first-line therapy, whereas more conservative options such as physical therapy and exercise are recommended for complex degenerative tears that may not respond well to repair [4].

Although meniscal tears are becoming more common, not all meniscal tears are symptomatic [10,11]. An *imaging* study of 991 volunteer subjects between the ages of 50 and 90 in Framingham, MA found that 31 ​% had meniscal tear on MRI but the majority (180 of 297 (61 ​%)) were in subjects who had not had any pain, aching, or stiffness in the previous month [1]. In contrast, a Swedish population-based study reported the annual incidence of *clinically diagnosed meniscal tears* as only 79 per 100,000 people (95 ​% CI [63, 94]) or 0.0079 ​% [11]. These observations suggest that meniscal tear is a much more common imaging finding than clinical diagnosis. A review of the literature suggested that the weighted average of meniscal tear prevalence on imaging in adults was 34.6 ​% (range 30–46 ​%) in asymptomatic cohorts, 75.6 ​% (range 68–87 ​%) in symptomatic cohorts, and 42.0 ​% (range 31–61 ​%) in mixed cohorts (Table 1) [1,[12], [13], [14], [15], [16], [17], [18], [19], [20], [21], [22]].

**Table 1:** Meniscal tear findings on MRI.

First Author	Year	Population	MRIs	Mean Age (SD)	Age Range	% Meniscal Tear on MRI	Source

**Asymptomatic**							
Horga	2020	Uninjured sedentary adults	230	44 (−)	18+	30 ​%	21
Davies-Tuck	2008	Postmenopausal, healthy women	74	57.0 (5.15)	50+	46 ​%	13
Davies-Tuck	2008	Postmenopausal, healthy women	20	60.7 (5.5)	–	45 ​%	12
**Symptomatic**							
Deshpande	2016	Adult patients with no previous MRI	84	64 (9)	45+	74 ​%	15
Sheridan	2021	Elderly patients	109	67 (6.16)	59–88	68 ​%	19
Shrestha	2024	Elderly patients	85	68.2 (1)	60–88	87 ​%	16
**Unselected**							
Ahmed	2021	Persons of any age	4466	45.38 (17.07)	–	42 ​%	20
Englund	2008	Community-based adults	991	62.3 (8.6)	50+	31 ​%	1
Kim	2011	Community-based elderly adults	358	71.8 (5.8)	50+	61 ​%	17

These observations raise questions about the mechanisms underlying knee pain in older persons with degenerative meniscal tear. Literature in this area suggests symptoms in patients with meniscal tear can be attributed to one or more of five mechanisms: mechanical, load-based, inflammatory, neoinnervation, and central sensitization.

### 1.2 Mechanical

Traditional teaching has suggested that mechanical symptoms—knee clicking, catching, locking, grinding—are associated with meniscal tears because unstable flaps or edges from the tear or meniscus fragments may impede or “obstruct” normal mobility and function [23,24]. However, research on patients who underwent knee arthroscopy found that preoperative mechanical symptoms were equally prevalent in patients both with and without a meniscal tear [25]. A 2013 sham-controlled randomized trial conducted in middle-aged persons with knee pain and meniscal tear showed that resection of the suspect tissue was not able to confer clinically significant pain relief or functional improvement over simple joint lavage [[26], [27], [28]]. A more recent literature review published in 2022 suggested that so-called mechanical symptoms appear to have modest sensitivity (range 0.32–0.69) and specificity (range 0.45–0.74) for both traumatic and degenerative meniscal tear [29]. Furthermore, a study of 565 consecutive patients (mean age 47.8, standard deviation 11.6) who underwent knee arthroscopy from 2012 to 2019 affirmed that knee catching, grinding, clicking, popping, and pain with pivoting were not significantly associated with stable or unstable meniscal tear, but were instead statistically significantly associated with cartilage damage [30]. These data suggest that for many individuals with catching, locking, grinding, or clicking, such mechanical symptoms arise from damaged cartilage rather than a torn meniscus [31,32].

### 1.3 Increased load

The meniscus is responsible for distributing load across the knee joint. In the healthy extended knee, approximately 40–60 ​% of the total load is borne by the menisci, with the medial meniscus responsible for about 50 ​% of the weight-bearing load in the medial tibiofemoral compartment and the lateral meniscus responsible for about 70 ​% in the lateral tibiofemoral compartment [33,34]. The load-bearing feature arises from the “hoop”/circumferential tension within the meniscus when forces are exerted across the knee [35]. Meniscal tears disrupt this load-bearing function significantly. One study of nine cadaver knees found that a posterior root tear in the medial meniscus resulted in a 25 ​% increase in peak contact pressure compared to a healthy knee [36]. Another study used a finite element model and found that a posterior horn tear in the medial meniscus resulted in a 121 ​% increase in shear stress of the meniscus under slight flexion simulation. Though meniscectomies are often used to treat symptomatic meniscal tears, they have also been linked to abnormal load bearing across the knee joint. A total lateral meniscectomy, for example, results in an increase in contact stress between 200 and 300 ​% of the normal range [34]. This persistent loss of load-bearing capacity may accelerate cartilage damage, leading to the development of osteoarthritis as well as osteonecrosis of the knee and insufficiency fractures [31,[37], [38], [39], [40], [41]]. All of these downstream effects will cause pain independent of the meniscal tear and support not only a decrease in meniscectomies but also greater research focused on surgical innovations to preserve degenerative meniscal tissue [36,42,43].

### 1.4 Inflammation

**Fig. 3:**
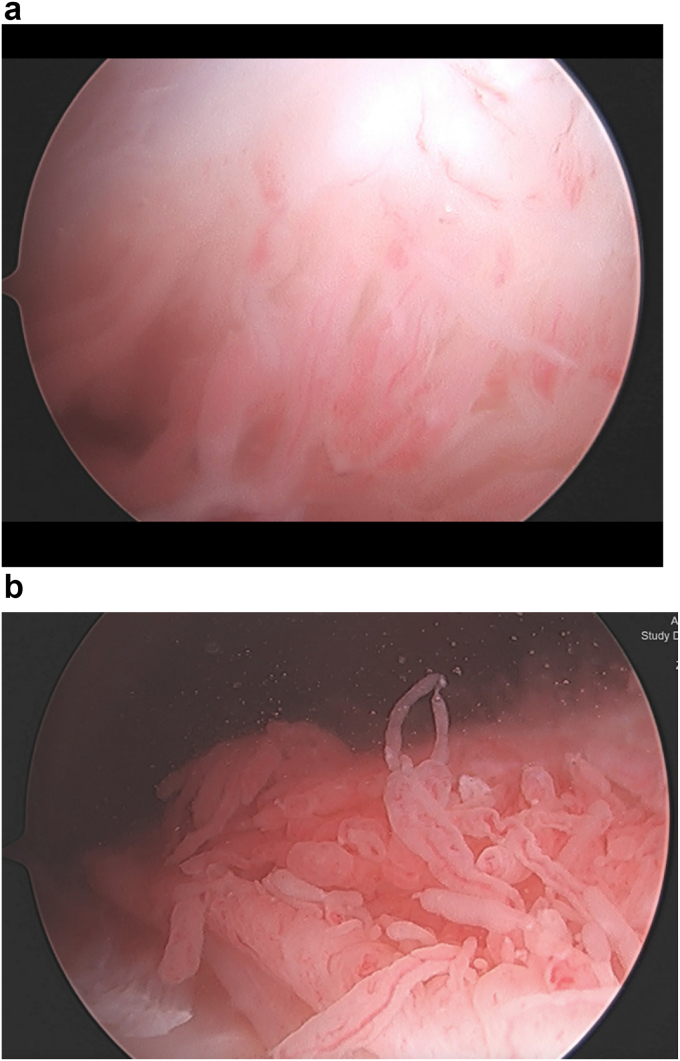
a. Image of mild meniscal synovitis. b. Image of severe meniscal synovitis.

The third mechanism through which meniscal tears can create pain is synovial inflammation, which manifests as synovitis and joint effusion. In the healthy knee joint, the meniscus receives most of its nutrients through synovial fluid due to its relative avascularity [3]. Injury to the meniscus and increased stress on its neighboring structures can result in varying levels of synovial tissue irritation (Fig. 3) [10,44]. A study using a combined sample from the Framingham Osteoarthritis and the MOST (Multicenter Osteoarthritis Study) cohorts found that joint effusion was present in 44.9 ​% of K/L grade 0 knees with meniscal damage (n ​= ​296) as compared with 30.6 ​% of knees in the same sample that did not have meniscal damage (adjusted odds ratio 1.8 (95 ​% CI [1.4, 2.4])) [10]. In a study of patients undergoing arthroscopic partial meniscectomy, the majority of patients demonstrated synovial mononuclear cell infiltration and synovial fibrosis [45]. Another study of 33 patients without knee OA undergoing arthroscopic partial meniscectomy found that 43 ​% had synovial inflammation; moreover, this inflammation was associated with preoperative pain and function scores independent of cartilage pathology [50]. Synovial inflammation is associated with knee pain; in a cohort of study participants aged 50 to 79 who either had OA or were at high risk of developing OA, synovitis conferred a 9.2-fold increase in the probability of moderate-to-extensive knee pain for those with a lot/extensive synovitis compared to those with no/questionable synovitis [[46], [47], [48], [49]]. Other studies investigating methods of managing inflammation with therapeutics such as platelet-rich plasma or docosapentaenoic acid have found positive short-term results, further suggesting that meniscal tear pain is linked with inflammation [44,51,52]. Research directly measuring the association among meniscal tear incidence, inflammation, and pain would help to support this proposed pathway.

### 1.5 Meniscal neoinnervation

Pathologic nerve growth into normally aneural structures (i.e., neoinnervation) occurs when normal neurovascular function is disrupted, as occurs in knee OA [53]. Many cases of meniscal tear either precede or occur simultaneously with incident symptomatic OA, suggesting that a subset of pain arising concomitantly with a degenerative meniscal tear may be attributed to neoinnervation within OA. The community-based imaging study referenced earlier found that 35 ​% of all subjects had meniscal damage (i.e., meniscal tear or destruction), whereas 82 ​% of all subjects who had radiographic OA (definite osteophytes) had meniscal damage on MRI [1,54]. OA has been associated with decreased resistance to vascular invasion of both articular cartilage and meniscal structures [[55], [56], [57]]. Osteoarthritic vascularization and innervation is hypothesized to occur due to an imbalance between angiogenic and anti-angiogenic factors such as IL-18 and TGF-α [58]. In knee osteoarthritis, increases in pressure on subchondral bone and shear stress have been associated with increases in meniscal and osteochondral neoinnervation with pain-sensitive fibers [[59], [60], [61], [62], [63]]. This phenomenon has been confirmed in recent studies in both human and animal models of knee OA [53,57,[64], [65], [66], [67]]. These recent discoveries lead to the hypothesis that a component of the pain in persons with meniscal tear may come from OA-driven neoinnervation in the meniscus. Further work directly examining the connection among meniscal tear, OA, and neoinnervation is required to test this hypothesis.

### 1.6 Sensitization

The last mechanism through which meniscal tears may become symptomatic is through a process termed “sensitization.” Pain arising from sensitization is referred to as *nociplastic* pain to distinguish it from nociceptive pain (which arises from stimulation of pain-sensitive fibers at sites of tissue damage) [68]. In cases of sensitization, there is no pathology in the peripheral nerves *per se* [69]. Instead, nociplastic pain arises from aberrations in the ascending pathways that carry information from the site of tissue damage (e.g. the knee synovium) to the brain, and in the descending pathways from the brain back to the spinal cord.

We hypothesize that sensitization can occur in cases of meniscal tear and concomitant knee OA, thus contributing to the overall burden of pain. These aberrations of ascending and descending pain pathways can be quantified using quantitative sensory testing (QST) [70]. Though no research has been done linking abnormal QST outcomes to meniscal tear, a growing body of evidence has begun to develop the association between abnormal QST outcomes and knee OA. One study found that up to 29 ​% of individuals with OA have abnormalities on static QST protocols; up to 55 ​% of individuals exhibit abnormalities on dynamic QST protocols [71]. Regarding specific attributes of sensitization, people with OA often perform worse than healthy controls on pain pressure threshold and temporal summation measures [72]. These abnormalities appear clinically relevant, with enhanced facilitation of ascending pathways or diminished inhibition of descending pain pathways leading to greater pain [71,73,74]. Research evaluating sensitization in degenerative meniscal tear while controlling for OA is necessary to establish an independent association. Since many degenerative meniscal tears are eventually accompanied by OA, sensitization may nonetheless be an important contributor to pain in persons with meniscal tear [32]. Further research is recommended to directly associate concomitant meniscal tear and OA with sensitization.

## 2 Discussion and future directions

We reviewed literature to investigate five potential sources of symptoms in older persons with degenerative meniscal tear. While mechanical symptoms may arise from a traumatic meniscal tear (such as a bucket handle tear catching on the femur and impeding smooth movement), catching/grinding/popping can occur in both traumatic and degenerative tears. We find that these “mechanical” symptoms more likely reflect damage to cartilage than to meniscus in middle-aged and older persons [4]. We also find that load-bearing symptoms manifest when a tear compromises the load-bearing function of the meniscus, transferring force to the nociceptor-invested subchondral bone. Increased stress on articular cartilage accelerates OA development and further contributes to joint pain [75]. Inflammatory symptoms, on the other hand, may occur when fragments of torn meniscus incite an inflammatory process in the synovium [44,50]. Pain may also occur when this structure is newly innervated with nociceptive fibers, as has been observed in cases of symptomatic OA [3,66,76,77]. Finally, patients may develop sensitization or nociplastic pain in the presence of chronic pain from one or more of these mechanisms, as well as from concomitant OA. All five mechanisms may act singly or in combination to promote pain in persons with degenerative meniscal tear.

Physical therapy and/or exercise (e.g., aerobic, strength, and flexibility training) is currently the recommended first-line treatment for degenerative meniscal tear. The therapeutic advantage of knowing which mechanisms occur in a particular patient's presentation lies in further targeting treatment. Since mechanical symptoms in persons with degenerative meniscal tear are likely tied to underlying knee osteoarthritis rather than the tear itself, physicians are increasingly reconsidering the value of arthroscopic meniscal resection. Though not discussed in this paper, meniscal repair may be indicated for traumatic tears in younger persons because these lesions occurred in otherwise healthy tissue [9]. Growing awareness of the load-bearing role of the meniscus has already led to a 35–40 ​% decrease in partial meniscectomies in the United States between 2010 and 2020 [78]. Regarding the inflammatory pathway, nonsteroidal anti-inflammatory drugs and acetaminophen can be prescribed to reduce inflammation-induced pain; other options such as intra-articular injections have also been noted for their pain relief, although concerns about accelerated OA progression and efficacy duration may limit their practicality [79,80]. The management of neoinnervation and sensitization in meniscal tear are in their infancy, with further research required. Pharmaceuticals such as tricyclic compounds and serotonin-norepinephrine reuptake inhibitors are currently used to treat central sensitization in patients with chronic pain, with evidence of modest efficacy [81]. By considering five potential mechanisms underlying symptomatic degenerative meniscal tear, clinicians may be better able target or combine treatments. Researchers may investigate alternative treatment options (such as extracorporeal shockwave therapy or meniscal substitution) or improve current methods (such as using biologics to facilitate meniscal repair) [[82], [83], [84]].

In our narrative review, we uncovered potential difficulty in attributing a symptomatic knee to either knee OA or a degenerative meniscal tear. Further research should also aim to better distinguish the cause of symptoms found in patients with both conditions. Additionally, research on treatment of symptomatic meniscal tear would be enhanced by methods distinguishing which of these characteristics contribute to pain in particular patient presentations. That approach to mechanistic phenotyping may permit medical, rehabilitative, and certain surgical therapies such as meniscal repair or root repair to be targeted or developed to address the actual source(s) of pain. Through continued research into the potential causes of symptoms associated with meniscal tear, clinicians may better understand the sources of knee pain in middle-aged and older persons and provide appropriate guidance to patients.

## Author contributions

*Conception and design:* Katz, Tsai.

*Analysis and interpretation of the data:* Katz, Tsai.

*Drafting of the article*: Tsai.

*Critical revision of the article for important intellectual content:* All Authors.

*Final approval of the article:* All Authors.

*Obtaining of funding:* Katz.

*Collection and assembly of data:* Tsai.

## Role of the funding source

Supported by: NIH/NIAMS Grants: U01AR071658, P30AR079206.

The funding sources had no role in the study design, collection, analysis and interpretation of the data, writing of the manuscript, or the decision to submit the manuscript for publication.

## Declaration of competing interest

The authors do not have any relevant conflicts of interest.
